# Supplementary material for: Entomo-virological investigation during the epizootic outbreak of sylvatic yellow fever in Rio Grande do Sul, Brazil, between 2021 and 2022
Source: Mem Inst Oswaldo Cruz. 2026 Mar 9;121:e250169. doi: 10.1590/0074-02760250169 (PMC12971024; doi:10.1590/0074-02760250169)
Supplement: Supplementary material [file 1678-8060-mioc-121-e250169-s1.pdf]

TABLE

Details of mosquito collection areas in Rio Grande do Sul during the spring and summer seasons of 2021 and 2022

Municipality	Area	Coordinates	Mosquito collection date	NHP collection date (YFV result)	Mosquito sampling method and effort time
Pinhal da Serra (PDS)	PDS1<br>PDS2<br>PDS3<br>PDS4<br>PDS4	-27.845, -51.159<br>-27.874, -51.263<br>-27.889, -51.147<br>-27.889, -51.147<br>-27.889, -51.147	Feb 09, 2021<br>Feb 09, 2021<br>Feb 10, 2021<br>Feb 11, 2021<br>Feb 02,2022	Feb 09, 2021 (YFV+)<br>Jan 25, 2021 (YFV+)<br>Feb 05, 2021 (YFV+)<br>Feb 08, 2021 (YFV+)<br>Feb 08, 2021 (YFV+)	HLC: 16.75 hours (2021)<br>HLC: 7 hours (2022)
Vacaria (VAC)	VAC1<br>VAC2<br>VAC3	-28.307, -50.823<br>-28.181, -50.913<br>-28.170, -50.923	Feb 23, 2021<br>Feb 24, 2021<br>Feb 24, 2021	Without NHP sampling<br>Without NHP sampling<br>Feb 24, 2021 (YFV+)	HLC: 4.5 hours
Esmeralda (ESM)	ESM1<br>ESM2	-27.975, -51.056<br>-27.975, -51.056	Feb 25, 2021<br>Feb 03, 2022	Feb 25, 2021 (YFV+)	HLC: 2 hours (2021)<br>HLC: 16 hours (2022)<br>BG-Pro: 24 hours (2022)
Campestre da Serra (CDS)	CDS1<br>CDS2	-28.778, -51.092<br>-28.778, -51.082	Mar 02, 2021<br>Mar 02, 2021	Mar 11, 2021 (YFV+)<br>Mar 11, 2021 (YFV+)	HLC: 1.73 hours
André da Rocha (ADR)	ADR1	-28.585, -51.575	Mar 04, 2021	Mar 04, 2021 (YFV+)	HLC: 1.5 hours
Ipê (IPE)	IPE1<br>IPE2	-28.640, -51.260<br>-28.698, -51.176	Mar 03, 2021<br>Mar 03, 2021	Without NHP sampling<br>Without NHP sampling	HLC: 4.17 hours
Protásio Alves (PRA)	PRA1<br>PRA2	-28.707, -51.527<br>-28.696, -54.521	Mar 04, 2021<br>Mar 04, 2021	Without NHP sampling<br>Without NHP sampling	HLC: 3.22 hours
Rolante (ROL)	ROL1<br>ROL2<br>ROL3	-29.563, -50.446<br>-29.575, -50.471<br>-29.634,-50.505	Mar 15, 2021<br>Mar 16, 2021<br>Mar 16, 2021	Feb 19, 2021 (YFV+)<br>Without NHP sampling<br>Without NHP sampling	HLC: 2.62 hours
São José do Ouro (SJO)	SJO1	-27.780, -51.503	Mar 23, 2021	Without NHP sampling	HLC: 1.5 hours
Tupanci do Sul (TDS)	TDS1<br>TDS2	-27.950, -51,524<br>-27.908, -51.514	Mar 24, 2021<br>Mar 24, 2021	Without NHP sampling	HLC: 4.25 hours
Machadinho (MAC)	MAC1	-27.677, -51.717	Mar 25, 2021	Without NHP sampling	HLC: 1.75 hours
Porto Alegre (POA)	POA1<br>POA2	-30.191, -51.078<br>-30.193, -51.124	Apr 06, 2021<br>Apr 07, 2021	Mar 10, 2021 (YFV-)<br>Mar 15, 2021 (YFV+)	HLC: 6.47 hours
Dois Irmãos (DOI)	DOI1	-29.616, -51.066	Apr 08, 2021	Mar 28, 2021 (YFV-)	HLC: 2 hours
Jaquirana (JAQ)	JAQ1	-28.855, -50.350	Apr 13, 2021	Apr 04, 2021 (YFV+)	HLC: 1.5 hours
Santo Antônio das Missões (SAM)	SAM1	-28.400, -55.442	Oct 19, 2021	Without NHP sampling At this location, Almeida et al.[18,19] detected NHPs with antibodies against YFV, ILHV, and SLEV	HLC: 16 hours<br>BG-Pro: 24 hours
Bossoroca (BOS)	BOS1		Oct 20, 2021	Without NHP sampling	HLC: 8 hours
Derrubadas (DER)	DER1<br>DER2<br>DER3	-27.252, -53.959<br>-27.260, -53.977<br>-27.249, -53.899	Dec 15, 2021	Without NHP sampling	HLC: 24h<br>BG-Pro: 24 hours

NHP: non-human primate; YFV: yellow fever virus; YFV+: yellow fever virus positive sample; YFV-: yellow fever virus negative sample; HLC: human landing catch.
